# Crystal structure and Hirshfeld surface analysis of bis­[hydrazinium(1+)] hexa­fluorido­silicate: (N_2_H_5_)_2_SiF_6_

**DOI:** 10.1107/S2056989019012672

**Published:** 2019-09-20

**Authors:** Ali Ouasri, Fatima Lambarki, Ali Rhandour, Mohamed Saadi, Lahcen El Ammari

**Affiliations:** a Laboratoire de Physico-chimie des Matériaux Inorganiques, Université Ibn Tofail, Faculté des Sciences, BP 133, 14000 Kenitra, Morocco; b Centre Régional des Métiers de l’Education et de la Formation, Madinat Al Irfane, Souissi, BP 6210 Rabat, Morocco; c Laboratoire de Chimie Appliquée des Matériaux, Centre des Sciences des Matériaux, Faculty of Sciences, Mohammed V University in Rabat, Avenue Ibn Batouta, BP 1014, Rabat, Morocco

**Keywords:** Hydrazinium (1+), hexa­fluorido­silicate, X-ray diffraction, Hirshfeld surfaces, infrared bonds, crystal structure

## Abstract

In the title inorganic mol­ecular salt, (N_2_H_5_)_2_SiF_6_, the silicon atom at the centre of the slightly distorted SiF_6_ octa­hedron [range of Si—F distances = 1.6777 (4)–1.7101 (4) Å] lies on a crystallographic inversion centre. In the crystal, the ions are connected by N—H⋯N and N—H⋯F hydrogen bonds; the former link the cations into [010] chains and the latter (some of which are bifurcated or trifurcated) link the ions into a three-dimensional network. The two-dimensional fingerprint plots show that F⋯H/H⋯F inter­actions dominate the Hirshfeld surface (75.5%) followed by H⋯H (13.6%) and N⋯H/H⋯N (8.4%) whereas F⋯F (1.9%) and F⋯N/N⋯F (0.6%) have negligible percentages. The title compound is isostructural with its germanium-containing analogue.

## Abstract 2

The title compound is a salt formed by a hydrazinium (1+) cation and a hexa­fluorido­silicate anion inter­connected by N—H⋯N and N—H⋯F hydrogen bonds.

## Chemical context   

Hydrazinium hexa­fluorido­metalate compounds have been studied by X-ray diffraction, vibrational spectroscopy and thermal analyses: they have been found to exist with two different formulae: N_2_H_6_ *M*F_6_ (Kojić-Prodić *et al.*, 1971*a* ▸,*b* ▸; Frlec *et al.*, 1980 ▸; Golič *et al.*, 1980 ▸; Cameron *et al.*, 1983 ▸; Knop *et al.*, 1983 ▸; Ouasri *et al.*, 2002 ▸) and (N_2_H_5_)_2_ *M*F_6_ (Gantar & Rahten, 1988 ▸; Leban *et al.*, 1994 ▸) where *M* = Ga, Si, Ti, Zr and Hf. The name ‘hydrazinium hexa­fluorido­silicate’ has been applied to both compounds: N_2_H_6_SiF_6_ and (N_2_H_5_)_2_SiF_6_.
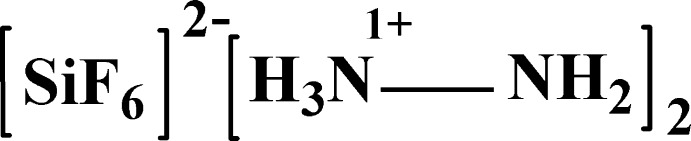


The crystal structure of N_2_H_6_SiF_6_ is well described by Frlec *et al.* (1980 ▸) and by Cameron *et al.* (1983 ▸), whereas that of (N_2_H_5_)_2_SiF_6_ has not previously been reported to our best knowledge. However, this compound was characterized by chemical analysis, vibrational spectroscopy and X-ray powder photography by Gantar & Rahten (1986 ▸), who determined the unit-cell parameters and the space group. We now describe the synthesis, single crystal structure and Hirshfeld surface analysis of the title compound, (I), at room temperature.

## Structural commentary   

**Figure 1:**
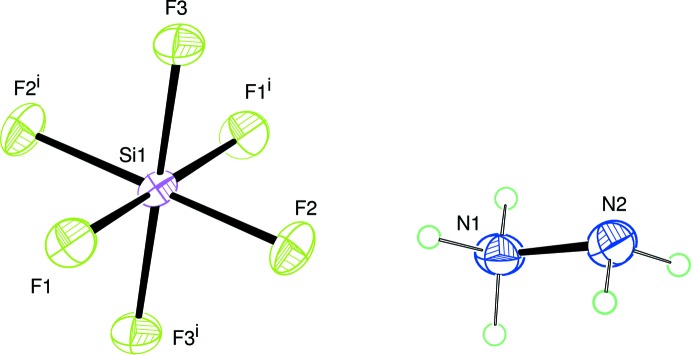
Mol­ecular structure of (I) with displacement ellipsoids drawn at the 50% probability level.

Compound (I) is an inorganic mol­ecular salt built up from N_2_H_5_^+^ cations and SiF_6_^2−^ anions, as shown in Fig. 1 ▸. In this structure, all atoms are in general positions except for the silicon atom, which is located at the Wyckoff position 2*d* on the inversion centre 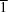 of the space group *P*2_1_/*n*. Thus, the silicon atom is connected to three unique fluorine atoms and their symmetry equivalents, forming a slightly elongated octa­hedron with Si—F distances in the range of 1.6777 (4) to 1.7101 (4) Å. The minimum and maximum *cis* F—S—F angles are 89.26 (2) and 90.74 (2)°, respectively. The N—N separation in the cation is 1.4416 (8) Å.

## Supra­molecular features and Hirshfeld surface analysis   

**Figure 2:**
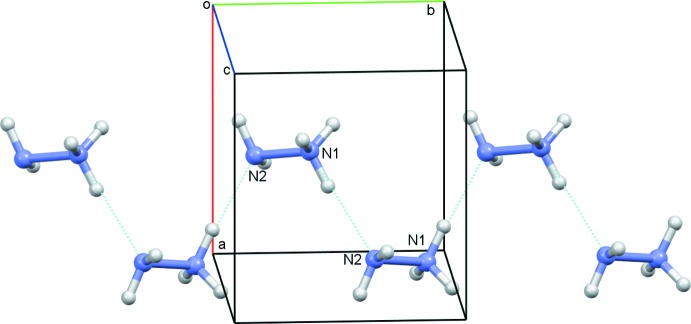
A view of the hydrazinium (1+) cations building an [010] chain through N—H⋯F hydrogen bonds (dashed blue lines).

**Figure 3:**
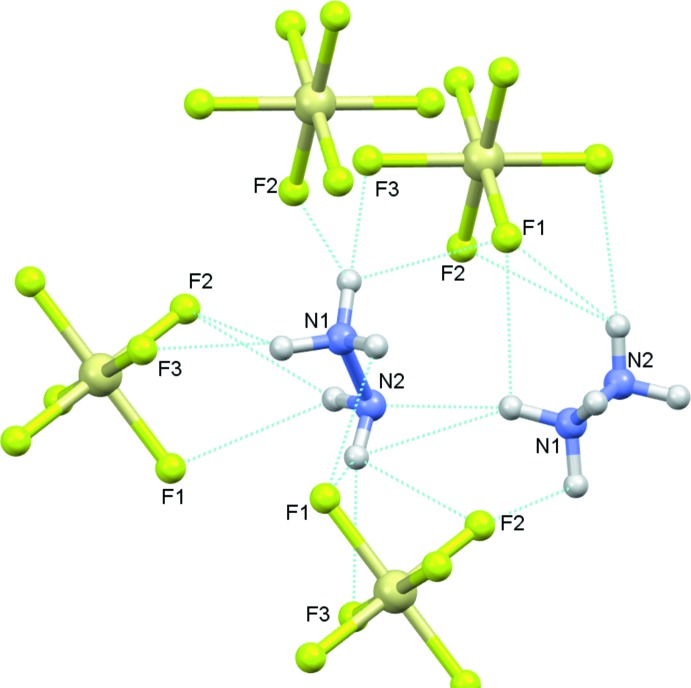
Details of the hydrogen bonds between the hydrazinium (1+) cations and (SiF_6_)^2−^ anions in (I).

**Figure 4:**
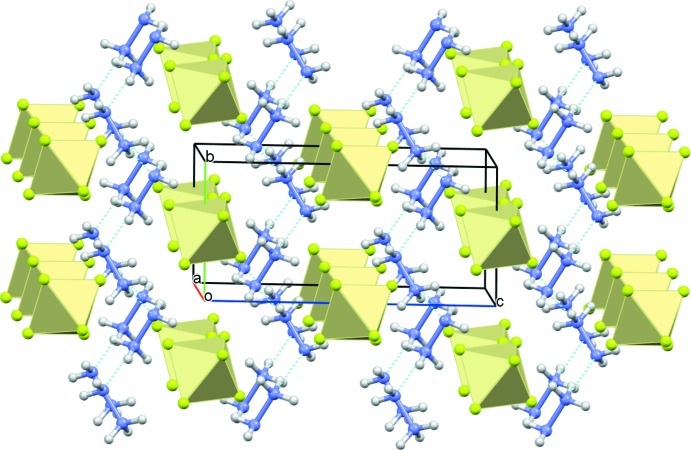
The crystal structure of (I) with the anions shown as polyhedra.

In the extended structure of (I), the hydrazinium cations are linked by strong N—H⋯N hydrogen bonds (Table 1 ▸), building an infinite zigzag chain propagating along the [010] direction as shown in Fig. 2 ▸. The [SiF_6_]^2−^ anion inter­acts with the (N_2_H_5_)^+^ cations through electrostatic attraction and accepts no fewer than ten simple, bifurcated or trifurcated N—H⋯F hydrogen bonds (Fig. 3 ▸, Table 1 ▸). This results in a three-dimensional network in which the hydrazinium cations build zigzag chains parallel to the *b*-axis direction and the [SiF_6_]^2−^ anions are stacked along the [100] direction (Fig. 4 ▸).

The packing of (I) was further investigated and qu­anti­fied with a Hirshfeld surface analysis (McKinnon *et al.*, 2004 ▸; Spackman & Jayatilaka, 2009 ▸) and two-dimensional fingerprint plots generated using the *CrystalExplorer* package (Turner *et al.*, 2017 ▸).

**Figure 5:**
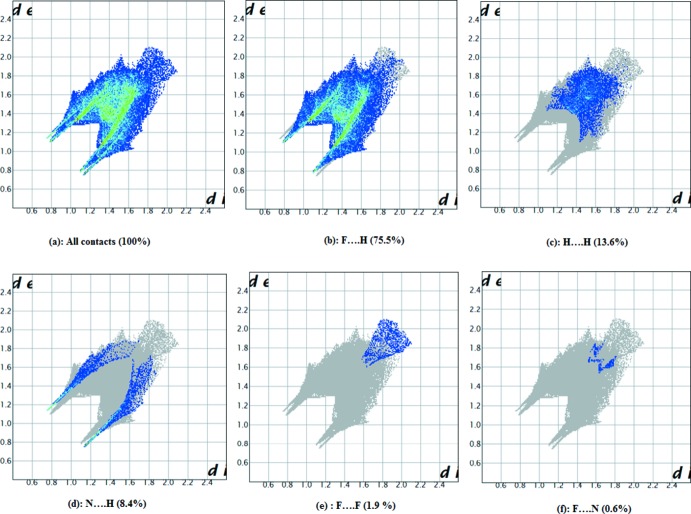
Fingerprint plots for the inter­actions present in the crystal packing of (I) showing (*a*) all contacts and those delineated into (*b*) F⋯H, (*c*) H⋯H, (*d*) N⋯H, (*e*) F⋯F and (*f*) F⋯N. The outline of the full fingerprint is shown in grey.

The acceptor atoms in the inter­actions are shown with negative electrostatic potentials (red regions), and donor atoms are shown with positive electrostatic potentials (blue regions). The N—H⋯F inter­actions in the structure are apparent from the relatively bright red-spots on the Hirshfeld surface of (I) mapped over *d*_norm_ (Fig. S1 in the supporting information). In order to provide qu­anti­tative information on the contribution of the inter­molecular inter­actions to the crystal packing, the three-dimensional *d*_norm_ surface is resolved into two-dimensional fingerprint plots, generated based on *d*_e_ and *d*_i_ distance scales and illustrated in Fig. 5 ▸(*a*)–(*f*) The F⋯H/H⋯F inter­actions appear as distinct spikes in the fingerprint plot, and occupy the majority of the total Hirshfeld surface (75.5%) as illustrated in Fig. 5 ▸(*a*); the characteristic ‘wingtip’ features indicate the N—H⋯F hydrogen bonds. The H⋯F inter­action are represented by a spike (*d*_i_ = 0.8, *d*_e_ = 1.1 Å) at the bottom left (donor), whereas the F⋯H inter­actions are represented by a spike (*d*_i_ = 1.1, *d*_e_ = 0.8 Å) at the bottom right (acceptor) of the fingerprint plot. The H⋯H contacts appear in the middle of the scattered points; these contacts comprise 13.6% of the total Hirshfeld surface [Fig. 5 ▸(*c*)]. The N⋯H contacts cover 8.4% of the total surface, as the third important contributor in the crystal packing, Fig. 5 ▸(*d*) while the F⋯F and F⋯N/N⋯F contacts make negligible contributions of 1.9% [Fig. 5 ▸(*e*)] and 0.6% [Fig. 5 ▸(*f*)], respectively.

## Database survey   

Hydrazinium (2+) hexa­fluorido­silicate, N_2_H_6_SiF_6_, at room temperature, crystallizes in a pseudo-tetra­gonal ortho­rhom­bic space group (*Pbca*, *Z* = 4), with *a* = 7.605 (1) Å, *b* = 7.586 (2) Å and *c* = 8.543 (1) Å (Frlec *et al.*, 1980 ▸; Cameron *et al.*, 1983 ▸). Its structure consists of centrosymmetric N_2_H_6_^2+^ and SiF_6_^2−^ ions arranged in a NaC1-type packing and connected by N—H⋯F hydrogen bonds, forming layers of cations and anions lying parallel to (001) plane.

Hydrazinium (1+) hexa­halogenometallates were studied by Gantar and co-workers (Gantar *et al.*, 1985 ▸; Gantar & Rahten, 1986 ▸) who showed that (N_2_H_5_)_2_GeF_6_ crystallizes in the monoclinic system, space group *P*2_1_/*n* (*Z* = 2), with cell parameters *a* = 6.015 (2) Å, *b* = 5.249 (1) Å, *c* = 11.181 (2)Å and β = 100.15 (2)° and is clearly isostructural with (I).

Fluoride complexes of titanium (IV) with ammonium cation derivatives include two hydrazinium hexa­fluorido­titanates (IV), (N_2_H_5_)_2_TiF_6_ (Leban *et al.*, 1994 ▸) and N_2_H_6_TiF_6_ (Kojić-Prodić *et al.*,1971*a* ▸,*b* ▸). The monoclinic crystals of (N_2_H_5_)_2_TiF_6_ [*P*2_1_; *Z* = 4; *a* = 7.815 (1) Å, *b* = 10.019 (1) Å, *c* = 9.338 (1) Å; β = 93.58 (1)°] exhibit racemic twinning but are not isostructural with (I). The crystal structure of (N_2_H_5_)_2_TiF_6_ consists of N_2_H_5_^+^ cations and two types of slightly distorted octa­hedral (TiF_6_)^2−^ anions. The N_2_H_5_^+^ cations and (TiF_6_)^2−^ anions are linked *via* N—H⋯F and N—H⋯N hydrogen bonds, building a three-dimensional network. Two other isostructural hydrazinium (l+) hexa­fluorido complexes, (N_2_H_5_)_2_ZrF_6_ and (N_2_H_5_)_2_HfF_6_, were prepared and characterized by chemical analysis, vibrational spectroscopy and X-ray powder diffraction (Gantar & Rahten, 1988 ▸). The infrared spectrum analysis of the title compound at room temperature confirms the obtained results by Gantar & Rahten with the exception of the assignments of two infrared bands (see supporting information).

## Synthesis and Infrared measurement technique.   

Hydrazinium (1+) hexa­fluorido­silicate (N_2_H_5_)_2_SiF_6_ crystals in the form of colourless blocks were obtained by slow evaporation, at room temperature, of an aqueous solution containing stoichiometric amounts of hydrazine NH_2_NH_2_ and H_2_SiF_6_. The infrared spectrum was recorded in the range 450–4000 cm^−1^ with a Vertex 70 FTIR spectrometer.

## Refinement   

Crystal data, data collection and structure refinement details are summarized in Table 2 ▸. H atoms were located in a difference-Fourier map and refined using a riding model with N—H = 0.86 Å and *U*_iso_(H) = 1.2*U*_eq_(N). The highest peak and the deepest hole in the final Fourier map are at 0.67 Å from F3 and 0.0 Å from Si1.

## Supplementary Material

Crystal structure: contains datablock(s) I. DOI: 10.1107/S2056989019012672/hb7850sup1.cif

Structure factors: contains datablock(s) I. DOI: 10.1107/S2056989019012672/hb7850Isup2.hkl

Supporting information file. DOI: 10.1107/S2056989019012672/hb7850sup3.pdf

Supporting information file. DOI: 10.1107/S2056989019012672/hb7850sup3.rtf

CCDC reference: 1953037

Additional supporting information:  crystallographic information; 3D view; checkCIF report

## Figures and Tables

**Table 1:** Hydrogen-bond geometry (Å, °)

*D*—H⋯*A*	*D*—H	H⋯*A*	*D*⋯*A*	*D*—H⋯*A*
N1—H1*A*⋯F1^i^	0.89	2.36	2.8436 (7)	114
N1—H1*A*⋯F3^ii^	0.89	2.04	2.9174 (7)	170
N1—H1*B*⋯N2^iii^	0.89	2.03	2.8963 (8)	165
N1—H1*C*⋯F3^iv^	0.89	2.02	2.9025 (7)	170
N1—H1*C*⋯F2^v^	0.89	2.49	2.9078 (7)	109
N2—H2*A*⋯F1^vi^	0.83	2.52	3.0952 (7)	127
N2—H2*A*⋯F2^vi^	0.83	2.64	3.0725 (7)	114
N2—H2*A*⋯F3^vi^	0.83	2.43	3.2373 (7)	167
N2—H2*B*⋯F3^vii^	0.86	2.49	3.2683 (7)	150
N2—H2*B*⋯F2^v^	0.86	2.51	3.1036 (7)	127
N2—H2*B*⋯F1^iv^	0.86	2.60	3.0124 (7)	110

Symmetry codes: (i) 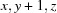; (ii) 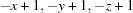; (iii) 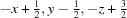; (iv) 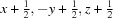; (v) 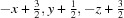; (vi) 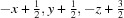; (vii) 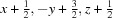.

**Table 2:** Experimental details

Crystal data	
Chemical formula	F_6_H_10_N_4_Si
*M*_r_	208.21
Crystal system, space group	Monoclinic, *P*2_1_/*n*
Temperature (K)	296
*a*, *b*, *c* (Å)	5.9496 (3), 5.2484 (2), 11.0029 (5)
β (°)	100.245 (1)
*V* (Å^3^)	338.10 (3)
*Z*	2
Radiation type	Mo *K*α
μ (mm^−1^)	0.42
Crystal size (mm)	0.31 × 0.24 × 0.16

Data collection	
Diffractometer	Bruker D8 VENTURE Super DUO
Absorption correction	Multi-scan (*SADABS*; Krause *et al.*, 2015 ▸)
*T*_min_, *T*_max_	0.638, 0.746
No. of measured, independent and observed [*I* > 2σ(*I*)] reflections	19683, 1488, 1379
*R*_int_	0.027
(sin θ/λ)_max_ (Å^−1^)	0.806

Refinement	
*R*[*F* ^2^ > 2σ(*F* ^2^)], *wR*(*F* ^2^), *S*	0.021, 0.059, 1.06
No. of reflections	1488
No. of parameters	54
H-atom treatment	H-atom parameters constrained
Δρ_max_, Δρ_min_ (e Å^−3^)	0.27, −0.22

Computer programs: *APEX3* and *SAINT-Plus* (Bruker, 2016 ▸), *SHELXT2014/7* (Sheldrick, 2015*a* ▸), *SHELXL2014/7* (Sheldrick, 2015*b* ▸), *ORTEP-3 for Windows* (Farrugia, 2012 ▸), *Mercury* (Macrae *et al.*, 2008 ▸)and *publCIF* (Westrip, 2010 ▸).

## supplementary crystallographic information

### Crystal data

**Table:**

F_6_H_10_N_4_Si	*F*(000) = 212
*M**_r_* = 208.21	*D*_x_ = 2.045 Mg m^−^^3^
Monoclinic, *P*2_1_/*n*	Mo *K*α radiation, λ = 0.71073 Å
*a* = 5.9496 (3) Å	Cell parameters from 1488 reflections
*b* = 5.2484 (2) Å	θ = 3.7–35.0°
*c* = 11.0029 (5) Å	µ = 0.42 mm^−^^1^
β = 100.245 (1)°	*T* = 296 K
*V* = 338.10 (3) Å^3^	Block, colourless
*Z* = 2	0.31 × 0.24 × 0.16 mm

### Data collection

**Table:**

Bruker D8 VENTURE Super DUO diffractometer	1488 independent reflections
Radiation source: INCOATEC IµS micro-focus source	1379 reflections with *I* > 2σ(*I*)
HELIOS mirror optics monochromator	*R*_int_ = 0.027
Detector resolution: 10.4167 pixels mm^-1^	θ_max_ = 35.0°, θ_min_ = 3.7°
φ and ω scans	*h* = −9→9
Absorption correction: multi-scan (SADABS; Krause *et al.*, 2015)	*k* = −8→8
*T*_min_ = 0.638, *T*_max_ = 0.746	*l* = −17→17
19683 measured reflections	

### Refinement

**Table:**

Refinement on *F*^2^	Hydrogen site location: mixed
Least-squares matrix: full	H-atom parameters constrained
*R*[*F*^2^ > 2σ(*F*^2^)] = 0.021	*w* = 1/[σ^2^(*F*_o_^2^) + (0.0348*P*)^2^ + 0.036*P*] where *P* = (*F*_o_^2^ + 2*F*_c_^2^)/3
*wR*(*F*^2^) = 0.059	(Δ/σ)_max_ = 0.001
*S* = 1.06	Δρ_max_ = 0.27 e Å^−^^3^
1488 reflections	Δρ_min_ = −0.22 e Å^−^^3^
54 parameters	Extinction correction: SHELXL-2018/3 (Sheldrick 2015b), Fc^*^=kFc[1+0.001xFc^2^λ^3^/sin(2θ)]^-1/4^
0 restraints	Extinction coefficient: 0.093 (10)

### Special details

**Table:**

Geometry. All esds (except the esd in the dihedral angle between two l.s. planes) are estimated using the full covariance matrix. The cell esds are taken into account individually in the estimation of esds in distances, angles and torsion angles; correlations between esds in cell parameters are only used when they are defined by crystal symmetry. An approximate (isotropic) treatment of cell esds is used for estimating esds involving l.s. planes.

### Fractional atomic coordinates and isotropic or equivalent isotropic displacement parameters (Å^2^)

**Table:**

	*x*	*y*	*z*	*U*_iso_*/*U*_eq_	
Si1	0.500000	0.000000	0.500000	0.01562 (7)	
F1	0.28419 (6)	−0.13785 (8)	0.55514 (4)	0.02516 (10)	
F3	0.31234 (7)	0.21784 (8)	0.42375 (4)	0.02473 (10)	
F2	0.55101 (7)	0.18926 (8)	0.62475 (4)	0.02576 (10)	
N1	0.46840 (9)	0.60149 (11)	0.77712 (5)	0.02307 (11)	
H1A	0.530893	0.639080	0.711612	0.035*	
H1B	0.334122	0.525916	0.752445	0.035*	
H1C	0.560407	0.497003	0.826736	0.035*	
N2	0.43562 (9)	0.83227 (11)	0.84285 (5)	0.02447 (11)	
H2A	0.390725	0.785787	0.906243	0.037*	
H2B	0.567906	0.898565	0.869296	0.037*	

### Atomic displacement parameters (Å^2^)

**Table:**

	*U*^11^	*U*^22^	*U*^33^	*U*^12^	*U*^13^	*U*^23^
Si1	0.01429 (10)	0.01983 (11)	0.01242 (10)	0.00065 (6)	0.00149 (6)	−0.00092 (6)
F1	0.02032 (17)	0.0332 (2)	0.02288 (17)	−0.00450 (14)	0.00636 (13)	0.00188 (14)
F3	0.02217 (17)	0.02512 (18)	0.02510 (18)	0.00506 (13)	−0.00071 (13)	0.00348 (13)
F2	0.02585 (18)	0.0309 (2)	0.01981 (17)	0.00025 (14)	0.00210 (13)	−0.01005 (14)
N1	0.0199 (2)	0.0245 (2)	0.0237 (2)	−0.00006 (16)	0.00101 (16)	0.00587 (18)
N2	0.0222 (2)	0.0289 (2)	0.0220 (2)	−0.00171 (18)	0.00320 (17)	0.00167 (18)

### Geometric parameters (Å, º)

**Table:**

Si1—F2^i^	1.6777 (4)	N1—N2	1.4416 (8)
Si1—F2	1.6777 (4)	N1—H1A	0.8900
Si1—F1^i^	1.6785 (4)	N1—H1B	0.8900
Si1—F1	1.6785 (4)	N1—H1C	0.8900
Si1—F3^i^	1.7101 (4)	N2—H2A	0.8268
Si1—F3	1.7101 (4)	N2—H2B	0.8624
			
F2^i^—Si1—F2	180.0	F1^i^—Si1—F3	90.52 (2)
F2^i^—Si1—F1^i^	89.90 (2)	F1—Si1—F3	89.48 (2)
F2—Si1—F1^i^	90.10 (2)	F3^i^—Si1—F3	180.0
F2^i^—Si1—F1	90.10 (2)	N2—N1—H1A	109.5
F2—Si1—F1	89.90 (2)	N2—N1—H1B	109.5
F1^i^—Si1—F1	180.0	H1A—N1—H1B	109.5
F2^i^—Si1—F3^i^	90.74 (2)	N2—N1—H1C	109.5
F2—Si1—F3^i^	89.26 (2)	H1A—N1—H1C	109.5
F1^i^—Si1—F3^i^	89.48 (2)	H1B—N1—H1C	109.5
F1—Si1—F3^i^	90.52 (2)	N1—N2—H2A	105.6
F2^i^—Si1—F3	89.26 (2)	N1—N2—H2B	108.1
F2—Si1—F3	90.74 (2)	H2A—N2—H2B	104.3

Symmetry code: (i) −*x*+1, −*y*, −*z*+1.

### Hydrogen-bond geometry (Å, º)

**Table:**

*D*—H···*A*	*D*—H	H···*A*	*D*···*A*	*D*—H···*A*
N1—H1*A*···F1^ii^	0.89	2.36	2.8436 (7)	114
N1—H1*A*···F3^iii^	0.89	2.04	2.9174 (7)	170
N1—H1*B*···N2^iv^	0.89	2.03	2.8963 (8)	165
N1—H1*C*···F3^v^	0.89	2.02	2.9025 (7)	170
N1—H1*C*···F2^vi^	0.89	2.49	2.9078 (7)	109
N2—H2*A*···F1^vii^	0.83	2.52	3.0952 (7)	127
N2—H2*A*···F2^vii^	0.83	2.64	3.0725 (7)	114
N2—H2*A*···F3^vii^	0.83	2.43	3.2373 (7)	167
N2—H2*B*···F3^viii^	0.86	2.49	3.2683 (7)	150
N2—H2*B*···F2^vi^	0.86	2.51	3.1036 (7)	127
N2—H2*B*···F1^v^	0.86	2.60	3.0124 (7)	110

Symmetry codes: (ii) *x*, *y*+1, *z*; (iii) −*x*+1, −*y*+1, −*z*+1; (iv) −*x*+1/2, *y*−1/2, −*z*+3/2; (v) *x*+1/2, −*y*+1/2, *z*+1/2; (vi) −*x*+3/2, *y*+1/2, −*z*+3/2; (vii) −*x*+1/2, *y*+1/2, −*z*+3/2; (viii) *x*+1/2, −*y*+3/2, *z*+1/2.
