# Supplementary material for: Crystal structure and Hirshfeld surface analysis of bis­[hydrazinium(1+)] hexa­fluorido­silicate: (N2H5)2SiF6
Source: Acta Crystallogr E Crystallogr Commun. 2019 Sep 20;75(Pt 10):1507–10. doi: 10.1107/S2056989019012672 (PMC6775730; doi:10.1107/S2056989019012672)
Supplement: Supplementary file 3 [file e-75-01507-sup3.pdf]

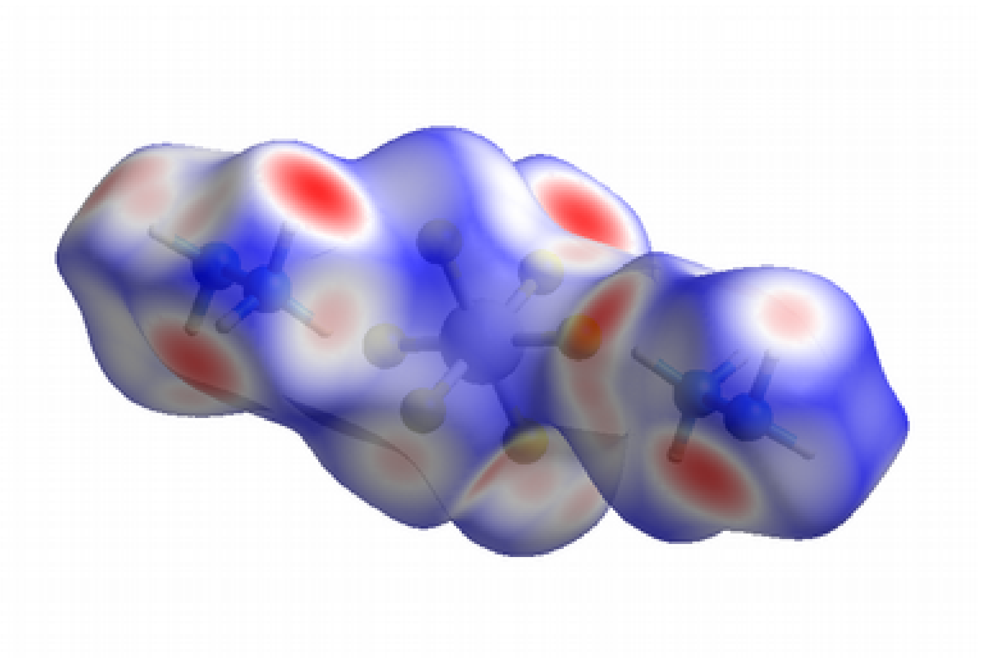

Figure 1S: Views of Hirshfeld surfaces mapped with d_{norm} properties of the $(N_2H_5)_2SiF_6$ compound.

Spectroscopic characterization of $(N_2H_5)_2SiF_6$

The Infrared spectrum of $(N_2H_5)_2SiF_6$ is given in supplementary material. The bands' assignment (supplementary material) is carried out based on previous works for homologous alkylammoniums compounds (Ouasri *et al.*, 2002, Gantar & Rahten, 1986). The cationic bands observed in 3300-2800 cm^{-1} and 1664- 974 cm^{-1} frequency ranges are due to N–H stretching modes, ν_{as} (NH_3)/$\nu_{as}(NH_2)$, ν_s (NH_3)/ν_s (NH_2) vibrations, r (NH_3) and ν (N—N) vibrations. The bands observed below 750 cm^{-1} are assigned to $(SiF_6)^{2-}$ internal vibrations. The free SiF_6^{2-} anions (O_h symmetry) possess the internal vibrational modes: $1A_{1g}$ (Ra) +$1E_g$ (Ra) + $1F_{2g}$ (Ra) + $2F_{1u}$ (IR) + $1F_{2u}$ (In). Inside the $(N_2H_5)_2SiF_6$ crystal ($P2_1/n$ centrosymmetric space group), the SiF_6^{2-} anions occupied C_i sites and, as a result, the A_{1g} [υ_1 (Si-F)], E_g [υ_2 (Si-F)] and F_{2g} [υ_5 (F-Si-F)] modes are expected to be only Raman active and not infrared active. The two bands observed at 726 and 651 cm^{-1} are due to F_{1u} [υ_3 (F-Si)] vibrations, while that observed at 474 and 435 cm^{-1} are assigned to F_{1u} [υ_4 (F-Si-F)] vibrations modes.

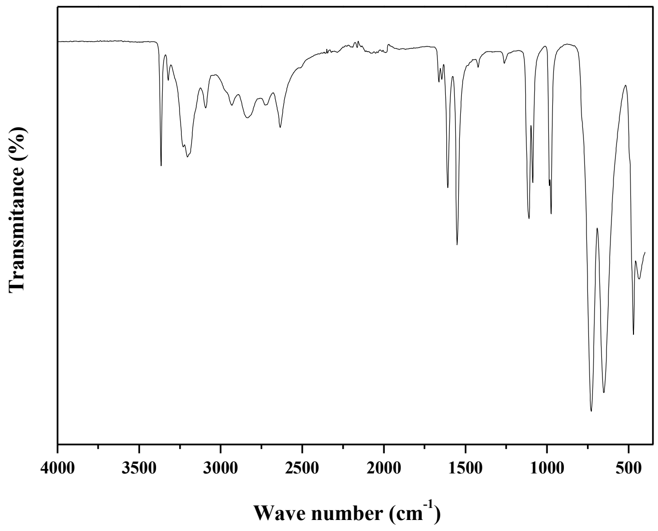

Figure 2s: Infrared spectrum of $(N_2H_5)_2SiF_6$ compound recorded at room temperature in the 400 - 4000 cm^{-1} spectral range.

Table 1s: Infrared bands assignments for $(N_2H_5)_2SiF_6$.

Infrared (cm^{-1})	Assignment
3368m	$\nu_{as}(NH_3)$

3323w	$\nu_{as}(NH_2)$
3235sh	$\nu_s(NH_3)$
3209m	$\nu_s(NH_2)$
3095w	$\delta_s(NH_3)$
2928w	$\delta_s(NH_2)$
2836w	Non fundamental modes
2722w	
2634m	
1664w	$\delta_{as}(NH_3)/\delta_{as}(NH_2)$
1613s	$\delta_{as}(NH_3)/\delta_{as}(NH_2)$
1550s	$\delta_s(NH_3)/\delta_s(NH_2)$
1259s	$r(NH_3)$
1113s	$r(NH_3)/\nu_{as}(N\text{-}N)$
1082m	$r(NH_3)/\nu_s(N\text{-}N)$
974s	$r(NH_3)$
726vs	$\nu_3(Si\text{-}F)$
651s	$\nu_3(Si\text{-}F)$
474m	$\nu_4(F\text{-}Si\text{-}F)$
435w	$\nu_4(F\text{-}Si\text{-}F)$

vs: very strong; s: strong; m: medium; w: weak; sh: shoulder
