# Supplementary material for: Crystal structure and Hirshfeld surface analysis of bis­[hydrazinium(1+)] hexa­fluorido­silicate: (N2H5)2SiF6
Source: Acta Crystallogr E Crystallogr Commun. 2019 Sep 20;75(Pt 10):1507–10. doi: 10.1107/S2056989019012672 (PMC6775730; doi:10.1107/S2056989019012672)
Supplement: Supplementary file 4 [file e-75-01507-sup3.rtf]

Figure 1S: Views of Hirshfeld surfaces mapped with dnorm properties of the (N2H5)2SiF6 compound.


 Spectroscopic characterization of (N2H5)2SiF6
The Infrared spectrum of (N2H5)2SiF6 is given in supplementary material. The bands' assignment (supplementary material) is carried out based on previous works for homologous alkyl­ammoniums compounds (Ouasri et al., 2002, Gantar & Rahten, 1986). The cationic bands observed in 3300-2800 cm-1 and 1664- 974 cm-1 frequency ranges are due to N–H stretching modes, nas (NH3)/nas(NH2), ns (NH3)/ns (NH2) vibrations, r (NH3) and n (N—N) vibrations. The bands observed below 750 cm-1 are assigned to (SiF6)2- internal vibrations. The free SiF62- anions (Oh symmetry) possess the internal vibrational modes: 1A1g (Ra) +1Eg (Ra) + 1F2g (Ra) + 2F1u (IR) + 1F2u (In). Inside the (N2H5)2SiF6 crystal (P21/n centrosymmetric space group), the SiF62- anions occupied Ci sites and, as a result, the A1g [u1 (Si-F)], Eg [u2 (Si-F)] and F2g [u5 (F-Si-F)] modes are expected to be only Raman active and not infrared active. The two bands observed at 726 and 651 cm-1 are due to F1u [u3 (F-Si)] vibrations, while that observed at 474 and 435 cm-1 are assigned to F1u [u4 (F-Si-F)] vibrations modes. 


Figure 2s: Infrared spectrum of (N2H5)2SiF6 compound recorded at room temperature in the 400 - 4000 cm-1 spectral range.


Table 1s: Infrared bands assignments for (N2H5)2SiF6.
Infrared (cm-1)	Assignment	
3368m	nas(NH3)	
3323w	nas(NH2)	
3235sh	ns(NH3)	
3209m	ns(NH2)	
3095w	ds(NH3)	
2928w	ds(NH2)	
2836w	
Non fundamental modes	
2722w		
2634m		
1664w	das(NH3)/d as (NH2)	
1613s	d as (NH3)/d as (NH2)	
1550s	ds(NH3)/ds(NH2)	
1259s	r(NH3)	
1113s	r(NH3)/nas(N-N)	
1082m	r(NH3)/ns(N-N)	
974s	r(NH3)	
726vs	n3(Si-F)	
651s	n3(Si-F)	
474m	n4(F-Si-F)	
435w	n4(F-Si-F)	
vs: very strong; s: strong; m: medium; w: weak; sh: shoulder
